# Association between Sjogren's Syndrome and Respiratory Failure: Put Airway, Interstitia, and Vessels Close Together: A National Cohort Study

**DOI:** 10.1371/journal.pone.0110783

**Published:** 2014-10-28

**Authors:** Jun-Jun Yeh, Hsuan-Ju Chen, Tsai-Chung Li, Yi-Sin Wong, Hsien-Chin Tang, Ting-Chun Yeh, Chia-Hung Kao

**Affiliations:** 1 Department of Family Medicine and Pulmonary Medicine, Ditmanson Medical Foundation Chia-Yi Christian Hospital, Chiayi, Taiwan; 2 Chia Nan University of Pharmacy and Science, Tainan, Taiwan; 3 Meiho University, Pingtung, Taiwan; 4 Management Office for Health Data, China Medical University Hospital, Taichung, Taiwan; 5 College of Medicine, China Medical University, Taichung, Taiwan; 6 Graduate Institute of Biostatistics, College of Management, China Medical University, Taichung, Taiwan; 7 Department of Healthcare Administration, College of Health Science, Asia University, Taichung, Taiwan; 8 Graduate Institute of Clinical Medical Science and School of Medicine, College of Medicine, China Medical University, Taichung, Taiwan; 9 Department of Nuclear Medicine and PET Center, China Medical University Hospital, Taichung, Taiwan; University of São Paulo School of Medicine, Brazil

## Abstract

**Objectives:**

Few studies have evaluated the association between Sjogren's syndrome (SS) and respiratory failure (RF). Thus, we conducted a retrospective national cohort study to investigate whether Sjogren's syndrome (SS) increases the risk of respiratory failure (RF).

**Methods:**

The cohort consisted of 4954 newly diagnosed patients with SS but without a previous diagnosis of RF, and 19816 patients as the comparison cohort from the catastrophic illnesses registry, obtained from the 2000–2005 period. All of the study participants were followed from the index date to December 31, 2011. We analyzed the association between the risk of RF and SS by using a Cox proportional hazards regression model, controlling for sex, age, and comorbidities.

**Results:**

The overall incidence rate of RF showed a 3.21-fold increase in the SS cohort compared with the comparison cohort. The adjusted HR of RF was 3.04 for the SS cohort compared with the comparison cohort, after we adjusted for sex, age, and comorbidities. The HRs of RF for patients with primary SS and secondary SS compared with the comparison cohort were 2.99 and 3.93, respectively (*P* for trend <.001). The HRs of RF increased as the severity of SS increased, from 2.34 for those with no inpatient care experience to 5.15 for those with inpatient care experience (*P* for trend <.001).

**Conclusion:**

This study indicates that clinical physicians should not only consider secondary SS but also primary SS as a critical factor that increases the risk of RF.

## Introduction

Respiratory failure (RF) remains a common reason for admission to intensive care units (ICUs) [1]. RF is a syndrome characterized by the failure of one or both gas exchange functions of the respiratory system, namely oxygenation and carbon dioxide elimination. In practice, RF is diagnosed when the arterial oxygen tension (PaO_2_) is <60 mmHg while breathing air (hypoxemia) or when the arterial carbon dioxide tension (PaCO_2_) is>50 mm (hypercapnia) [2]. The hypoxemia may be accompanied by hypercapnia. Furthermore, RF may be an acute RF (ARF) or a chronic RF.^2^ RF can arise from an abnormality in any of the components of the respiratory system, including the airways, alveoli, interstitia, and vessels [2], [3]. The mortality rate of ARF in critically ill patients is between 40% and 65% [4].

In the general population, Sjogren's syndrome (SS) affects approximately 3%–4% of adults and may be associated with a clinically significant impairment of a person's health and well-being [5]. SS has a broad clinical spectrum, extending from autoimmune exocrinopathy to extraglandular (systemic) diseases affecting the lungs, blood vessels, and lymph nodes [6]. In the absence of other autoimmune diseases, the syndrome is classified as primary SS [7]; when associated with other autoimmune diseases such as rheumatoid arthritis (RA), scleroderma, and systemic lupus erythematosus (SLE), it is classified as secondary SS. Moreover, SS is associated with various diseases of the thyroid gland, diabetes mellitus (DM), and hypertension [8], [9]. Despite being a benign autoimmune disease, SS can terminate in a lymphoid malignancy [10]. Previous studies have reported the development of respiratory diseases associated with the interstitial [10]–[13], alveoli [10], [13], [14], airways [10], [12], [13], [15], and vessels [10], [13], [15]–[18] of the lungs in patients with SS [13], [19]–[21]. Moreover, some studies have associated SS with pulmonary functional impairments, including an obstructive, restrictive, or mixed ventilation [6], [12], [19].

In recent years, several pathophysiological conditions have been associated with small airway diseases other than chronic obstructive pulmonary disease (COPD) and asthma, including airway infections such as pneumonia and connective tissue diseases such as SS [22]. Exacerbation of COPD, asthma, or pulmonary embolism (PE) [3] may contribute to RF. Moreover, SS may develop from subclinical [12], [23]–[25] to severe deteriorated [26] conditions, because of respiratory lesion such as chronic pulmonary fibrosis [12], [13], [19] and pulmonary arterial hypertension [3], [11], [13], [18], [27]. This is probably the first English literature research to address SS associated with RF in the general population.

## Materials and Methods

### Data source

Taiwan's National Health Insurance (NHI) is a universal insurance system established in 1996 by the Bureau of National Health Insurance of the Department of Health, and covers nearly 99% of the population in Taiwan. In this study, patient data from 2000 to 2011 were obtained from Taiwan's National Health Insurance Research Database (NHIRD), which contains each patient's information including sex, birthdate, and the registry of medical services. All personal information was encrypted before release to the public to protect patient privacy.

For this study, disease diagnosis was from disease records according to the International Classification of Diseases, 9th Revision, Clinical Modification (ICD-9-CM) in inpatient and catastrophic illnesses registry files.

### Data Availability Statement

All data and related metadata are deposited in an appropriate public repository: The study population's data were from Taiwan NHIRD (http://w3.nhri.org.tw/nhird//date_01.html) are maintained by Taiwan National Health Research Institutes (http://nhird.nhri.org.tw/). The National Health Research Institutes (NHRI) is a non-profit foundation established by the government.

### Ethics Statement

The NHIRD encrypts patient personal information to protect privacy and provides researchers with anonymous identification numbers associated with relevant claim information, including patients' sex, dates of birth, medical services utilized, and prescriptions. Patient consent is not required for accessing the NHIRD. This study was approved by the Institutional Review Board of China Medical University (CMU-REC-101-012). Our IRB specifically waived the consent requirement.

### Study population

We used a retrospective population-based cohort design. The cohort consisted of 4954 newly diagnosed patients with Sjögren syndrome (SS, ICD-9-CM code 710.2) but without a previous diagnosis of RF during 2000–2005 in the catastrophic illnesses registry, and the date of diagnosed SS was defined as the index date. Four participants in the comparison cohort for each patient with SS were randomly selected from insured people without a history of SS and RF, frequency-matched according to age (per 5 y), sex, and index-year. We thus included 4954 patients as the SS cohort and 19 816 patients as the comparison cohort. The primary SS diagnosis was based on the European Study Group on Classification Criteria for Sjogren's Syndrome, a revised version of the European criteria proposed by an American–European study [7]; therein, a secondary SS was defined as a diagnosis of SS in patients with a previous diagnosis of rheumatoid arthritis (RA; ICD-9-CM code 714), systemic lupus erythematosus (SLE; ICD-9-CM code 710.0), scleroderma (ICD-9-CM code 710.1), or primary biliary cirrhosis (PBC; ICD-9-CM code 571.6).

We identified the following as comorbidities before the index date: pneumonia (ICD-9-CM code 480-487), asthma (ICD-9-CM code 493 and 494), hypertension (ICD-9-CM code 401-405), diabetes mellitus (DM, ICD-9-CM code 250), chronic obstructive pulmonary disease (COPD, ICD-9-CM code 491, 492 and 496), pulmonary embolism (PE, ICD-9-CM code 415.1, 639.6 and 673.8), thyroid disease (ICD-9-CM code 240-242 and 244-246), and lymphoma (ICD-9-CM code 200-208).

We identified the first diagnosis of RF (ICD-9-CM 518.81, 518.83, and 518.84) by using hospitalization records as the study endpoint. All of the study participants were followed from the index date to the study endpoint, December 31, 2011, or when the patient withdrew from the insurance system or died.

### Statistical analysis

Demographic data, including age (<45, 45–59, and ≥60), sex, and comorbidity, were compared between patients with and without SS by using the chi-square test for categorical variables and the Student's t-test for continuous variables. To estimate the cumulative incidence of RF risks according to SS status, we performed survival analysis by using the Kaplan–Meier method, and significance was determined using the log-rank test. Multivariate Cox proportional hazard regression was used to examine the effect of SS on the risk of RF, which was determined based on the adjusted hazard ratio (HR) with a 95% confidence interval (CI). The multivariable model was used to adjust for age, sex, and comorbidities of pneumonia, asthma, hypertension, DM, COPD, PE, thyroid disease, and lymphoma.

All statistical analyses were performed using the SAS 9.3 statistical package (SAS Institute Inc., NC, USA), and R software (R Foundation for Statistical Computing, Vienna, Austria) was used to plot Kaplan–Meier survival curves; *P*<.05 for 2-tailed tests was considered significant.

## Results

In this study, we evaluated 4954 SS patients and 19 816 individuals without SS with a similar average (53 y) and sex ratio (female: 87.08%; Table 1). The SS cohort had a higher proportion of comorbidities, such as pneumonia, asthma, hypertension, DM, COPD, PE, thyroid disease and lymphoma, than did the comparison cohort.

**Table 1:** Baseline demographic factors and comorbidity of study participants according to sjogren's syndrome status.

	Control N = 19,816		SS N = 4,954		p-value
Characteristics	N	%	n	%	
Gender					0.99
Female	17,256	87.08	4,314	87.08	
Male	2,560	12.92	640	12.92	
Age, years					0.99
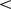 45	5,548	28.00	1,387	28.00	
45–59	7,772	39.22	1,943	39.22	
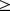 60	6,496	32.78	1,624	32.78	
Mean (SD)	53.32	(14.45)	53.43	(14.36)	0.66
Comorbidity					
Pneumonia	460	2.32	205	4.14	<0.001
Asthma	267	1.35	122	2.46	<0.001
Hypertension	1,516	7.65	473	9.55	<0.001
Diabetes mellitus	909	4.59	190	3.84	0.02
COPD	306	1.54	120	2.42	<0.001
Pulmonary embolism	6	0.03	13	0.26	<0.001
Thyroid disease	203	1.02	169	3.41	<0.001
Lymphoma	15	0.08	15	0.30	<0.001

Abbreviation: COPD, chronic obstructive pulmonary disease; SS, sjogren's syndrome; SD, standard deviation

**Figure 1:**
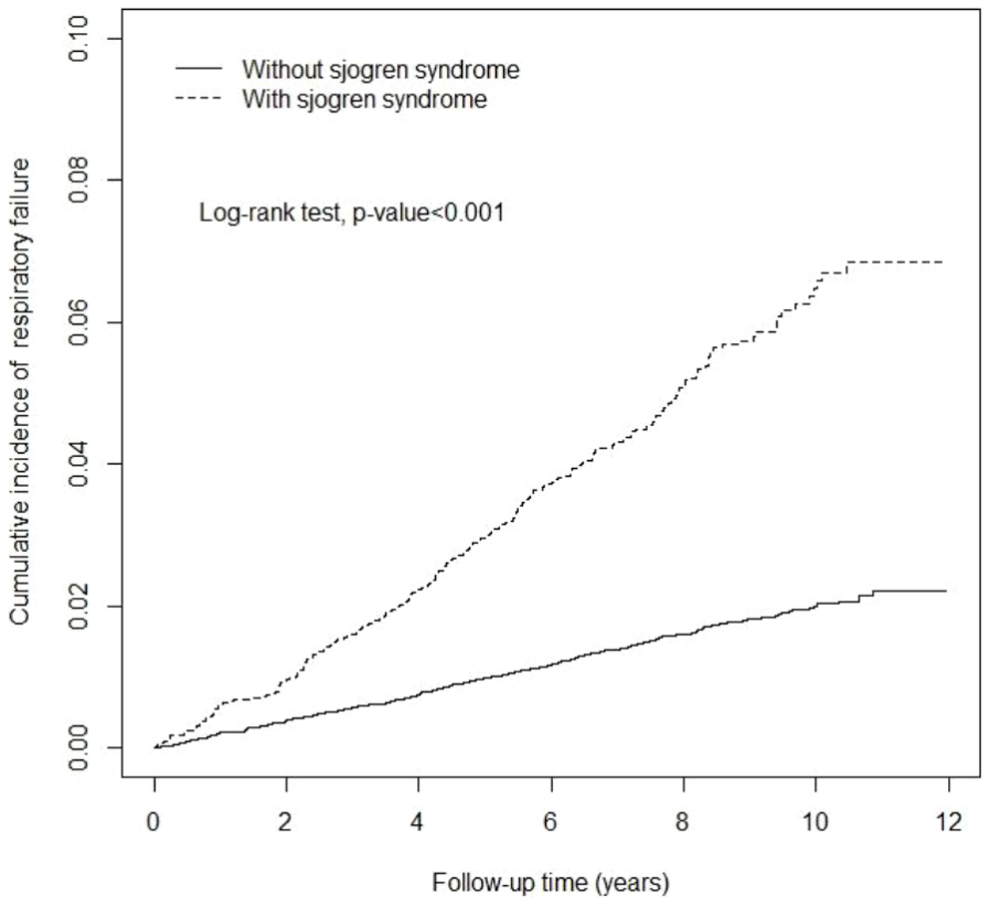
Cumulative incidence curves of respiratory failure for Sjogren syndrome and control Groups.

The Kaplan–Meier analysis showed that the cumulative incidence curves of RF were significantly higher in the SS cohort than in the comparison cohort (log-rank test *P*<.001) (Figure 1). The overall incidence rate of RF showed a 3.21-fold increase in SS cohort compared with the comparison cohort (6.44 vs 2.01 per 1000 person-y). The adjusted HR of RF was 3.04 (95% CI = 2.57–3.59) for the SS cohort compared with the comparison cohort, after we adjusted for sex, age, and comorbidities (Table 2). In addition, we observed that the death number of discharged in hospitalized patients of respiratory failure were 118 (45.46%) and 0 (0.00%) in the SS and non-SS cohorts (data not show).

**Table 2:** Incidence rates and hazard ratio for respiratory failure according to sjogren's syndrome status stratified by demographic factors and comorbidity.

	Sjogren's syndrome						Compared to non-sjogren's syndrome			
	No			Yes			Crude HR (95% CI)		Adjusted HR (95% CI)	
Characteristics	Case	Person-years	IR	Case	Person-years	IR				
Overall	317	157919.10	2.01	254	39421.58	6.44	3.21	(2.72–3.78)***	3.04	(2.57–3.59)***
Gender										
Female	277	139081.19	1.99	177	34726.87	5.10	2.56	(2.12–3.09)***	2.45	(2.02–2.96)***
Male	40	18837.91	2.12	77	4694.71	16.40	7.75	(5.29–11.36)***	7.10	(4.83–10.45)***
Age, years										
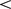 45	91	46049.32	1.98	22	11793.58	1.87	0.94	(0.59–1.50)	1.03	(0.63–1.68)
45–59	139	63770.04	2.18	44	15867.92	2.77	1.27	(0.91–1.79)	1.24	(0.88–1.76)
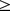 60	87	48099.74	1.81	188	11760.08	15.99	8.84	(6.86–11.40)***	8.29	(6.42–10.70)***
Comorbidity status‡										
No	187	141523.81	1.32	133	32877.82	4.05	3.06	(2.45–3.83)***	3.11	(2.49–3.88)***
Yes	130	16395.29	7.93	121	6543.76	18.49	2.34	(1.83–3.00)***	2.25	(1.75–2.89)***

Sex-specific analysis revealed that the incidence rates of RF in females and males with SS were 5.10 and 16.40 per 1000 person-years, respectively, which are higher than those in the comparison cohort (1.99 and 2.12 per 1000 person-y, respectively). In addition, compared with the comparison cohort, females exhibited a 2.45-fold (adjusted HR = 2.45, 95% CI = 2.02–2.96) higher risk of developing RF, and males showed a 7.10-fold (adjusted HR = 7.10, 95% CI = 4.83–10.45) higher risk (*P* for interaction <.001). Compared with comparison cohort, the greatest magnitude of HR was observed in patients with SS aged 60 years and older (8.29, 95% CI = 6.42–10.70). Regardless of the participants' comorbidities, SS patients had a higher HR of RF than the comparison cohort (Table 2).

Table 3 lists the combined effects of SS and comorbidities including pneumonia, hypertension, DM, and COPD on the risk of RF compared with the referent group of no SS and no corresponding comorbidity. We observed a greater magnitude of HRs of RF for patients with SS and pneumonia, hypertension, DM, and COPD compared with patients with no SS and no counterpart comorbidity (HR = 12.54, 95% CI = 8.91–17.65; 11.72, 8.89–15.44; 14.30, 10.12–20.20; and 15.19, 10.26–22.49, respectively).

**Table 3:** Joint effect of SS and comorbidity in association with respiratory failure in study population.

Variable		Case	IR	HR (95% CI)
SS	Pneumonia			
No	No	287	1.85	1.00
No	Yes	30	10.81	4.52 (3.09–6.61)***
Yes	No	216	5.68	3.09 (2.59–3.68)***
Yes	Yes	38	27.93	12.54 (8.91–17.65)***
SS	Hypertension			
No	No	226	1.53	1.00
No	Yes	91	9.34	4.85 (3.75–6.27)***
Yes	No	180	4.97	3.29 (2.71–4.01)***
Yes	Yes	74	22.85	11.72 (8.89–15.44)***
SS	Diabetes			
No	No	260	1.71	1.00
No	Yes	57	10.03	4.58 (3.41–6.14)***
Yes	No	216	5.65	3.32 (2.77–3.97)***
Yes	Yes	38	31.73	14.30 (10.12–20.20)***
SS	COPD			
No	No	285	1.82	1.00
No	Yes	32	18.26	6.62 (4.52–9.67)***
Yes	No	225	5.82	3.21 (2.69–3.82)***
Yes	Yes	29	39.26	15.19 (10.26–22.49)***

Abbreviation: SS, sjogren syndrome; IR, incidence rate, per 10,000 person-years; HR, hazard ratio; CI, confidence interval; COPD, chronic obstructive pulmonary disease.

Multivariate-adjusted model including gender, age, and comorbidities.

*** p<0.001.

The HRs of RF for patients with primary and secondary SS compared with the comparison cohort were 2.99 (95% CI = 2.52–3.54) and 3.93 (95% CI = 2.40–6.41), respectively (*P* for trend <.001) (Table 4). Overall, the HRs of RF increased as the severity of SS increased, from 2.34 (95% CI = 1.92–2.85) for those with no inpatient care experience to 5.15 (4.13–6.42) for those with inpatient care experience compared with the comparison cohort (*P* for trend <.001).

**Table 4:** Incidence and adjusted hazard ratio of respiratory failure in different subgroups and severity sjogren syndrome.

	N	Cases	IR	Crude HR (95% CI)	Adjusted HR (95% CI)
Without sjogren's syndrome	19816	317	2.01	1.00 (Reference)	1.00 (Reference)
Subgroups of sjogren's syndrome					
Primary sjogren syndrome	4672	237	6.37	3.17 (2.68–3.76)***	2.99 (2.52–3.54)***
Secondary sjogren syndrome‡	282	17	7.60	3.78 (2.32–6.16)***	3.93 (2.40–6.41)***
Sjogren syndrome					
Hospital admissions					
No	3,743	147	4.92	2.45 (2.01–2.98)***	2.34 (1.92–2.85)***
Yes	1,211	107	11.23	5.60 (4.50–6.97)***	5.15 (4.13–6.42)***

Abbreviation: IR, incidence rate, per 1,000 person-years; HR, hazard ratio; CI, confidence interval.

Adjusted HR: adjusted for age, gender, and comorbidity in Cox proportional hazards regression

‡ A secondary SS was defined a diagnosis of SS in patients with a previous diagnosis of rheumatoid arthritis (ICD-9-CM code 714), systemic lupus erythematosus (SLE; ICD-9-CM code 710.0), scleroderma (ICD-9-CM code 710.1), or primary biliary cirrhosis (ICD-9-CM code 571.6).

*** p<0.001.

## Discussion

The initial findings of this study suggest that the incidence of RF in the primary or secondary SS cohort was higher than that in the non-SS cohort, regardless of sex, age, or comorbidities. In Papathanasiou et al [12], compared with the control participants, the incidence of pulmonary abnormalities in SS was nonsignificant and clinically negligible. By contrast, Strimlan et al [20] observed chest lesions, such as diffuse interstitial fibrosis, recurrent pneumonitis, pleural effusions, and suspected lymphoma or pseudolymphoma, in patients with SS.

The pathophysiology of RF includes the impairment of the airways, alveoli, interstitia, and vessels of the lungs. Further deterioration of the respiratory lesions of SS may lead to progressive changes such as airway obstruction [6], [28], [29], alveolitis [24], [30], pulmonary lymphoma [20], interstitial fibrosis [20], vasculitis [13], or pulmonary arterial hypertension [17], [18]. This indicates a positive association of SS with the airways, alveoli, interstitia, and vessels of the lung, which in turn may contribute to the incidence of RF, even without comorbidities [28], [31], [32].

In our study, the SS cohorts with pneumonia [12], [22], hypertension [9], [33], DM [8], or COPD [29] exhibited increased risks of RF. In addition, the SS cohort with COPD exhibited the highest IR and HR, and the SS cohort with patients aged ≥60 years exhibited a higher RF incidence than did the non-SS cohort; moreover, concordant findings were observed in other studies investigating elderly patients with comorbidities such as hypertension, DM, or COPD [9], [29]. The therapeutic management of the SS cohort with elderly patients could have been complicated by comorbidities and an increased rate of adverse events related to the applied therapeutic agents and polypharmacy. Therefore, effective management of comorbidities, such as COPD, and careful follow-up are necessary for SS patients [34].

The primary SS cohort exhibited a higher incidence of RF than that in the non-SS cohort. Based on Papiris et al, the airway epithelia of the lungs may be the main target of inflammatory lesions in patients with primary Sjogren's syndrome, which may be a common subclinical condition that leads to obstructive small airway physiological abnormalities [35]. Moreover, primary Sjogren's syndrome associated with interstitial lung disease [36] and pulmonary arterial hypertension [18] were the 2 factors that contributed to the development of RF. Furthermore, based on Constantopoulos et al, the cumulative effects of diffuse interstitial lung disease contribute the most to RF in primary SS (25%), followed by small airway disease (22%), desiccation of the upper respiratory tract (17%), and large airway obstruction (8%) [21], [28].

Based on the non-SS cohort as a reference, the incidence of RF in the secondary SS cohort exhibited the highest IR and HR. Moreover, a previous study reported a higher incidence of respiratory lesions in the secondary SS cohort than that in the primary SS cohort, which agrees with our findings [37]. Furthermore, another study [12] reported a higher incidence of obstructive airway disease in the secondary SS cohort than in the primary SS cohort, and that obstructive airway disease can contribute to the development of RF [38].

No studies have indicated an association between the frequency of admission and the incidence of RF. In our study, the frequency of admission was higher in the SS cohort than in the non-SS cohort. The patients in the SS cohort at admission may present with RF accompanied by an acute exacerbation of pulmonary fibrosis [39] or pulmonary emboli [40], which may contribute to pulmonary arterial hypertension [26], [38], [39]. The risk of RF increases with the number of SS exacerbations and admissions [41]. These findings imply a positive association between the incidence of RF and recurrent airway inflammation [29], [42], interstitial fibrosis [11], [41], alveolitis [43], and chronic pulmonary arterial hypertension [18], [40], all of which may cause impairment or damage the airways [28], [42], interstitia/aleveoli [11], [41], [43], and pulmonary vessels [40]. These findings indicate poor control as a critical factor for the incidence of RF in patients with SS [19], [28], [44], [45].

The clinical presentation of SS may vary. The onset is insidious [12], [43] and usually begins in women aged 40–60 years; however, it can also affect men and children [46]. The initial manifestations of SS may include asthma, COPD [29], or pneumonia [20], [21], which may contribute to the diagnosis of SS [20], [21], [47]. Concurrently, the subclinical manifestations of SS may delay the diagnosis [23], [43], [48]. Thus, the initial symptoms of primary SS can be overlooked or misinterpreted easily, and diagnosis can be delayed for several years. Therefore, this study emphasizes the importance of early diagnosis of SS and put the airway, interstitia and vessels close together in the development of RF among the patients with primary SS or secondary SS.

### Limitations

Several limitations must be considered when interpreting the findings of this study. The NHIRD provides no detailed lifestyle information, such as smoking, body mass index, and physical activity, all of which were potential confounding factors for this study. However, the treatment and lifestyle modifications of patients with SS may implicate these factors in the accelerated development of respiratory lesions in SS. In addition, our study data provided no information on the SS severity scale, including disease activity, functional impairment, and physical damage [49]. Another limitation was the lack of individual information of drugs' use in the database, including pilocarpine, hydroxychloroquine, and glucocorticosteroids, which were the possible risk factors for respiratory failure could have been used to adjust for the outcomes of interest. However, Taiwan launched a national health insurance (NHI) in 1995, operated by a single-buyer, the government. Medical reimbursement specialists and peer review should scrutinize all insurance claims. Therefore, under the reimbursement coverage of NHI, every patient with SS in this study should already receive the standard treatments for SS including systemic steroid or other immunosuppressive drugs. Despite our meticulous study design to control for the confounding factors, a key limitation of this study is the potential for bias caused by possible unmeasured or unknown confounders.

### Strengths

The strength of this study is that it provides a nationwide, population-based, longitudinal cohort study on the risk of RF in Asian patients with SS. The outcomes of the cohort study in the general population may be similar to those in the “real world”. [50] Thus, the findings of this study can be generalized to the general population.

## Conclusion

In conclusion, this nationwide study investigating approximately 4954 patients with SS with 19 816 follow-up person-years reveals that patients with SS exhibit a 3.04-fold increased risk of developing RF compared with the general population. These findings highlight the importance of a multidisciplinary team adopting an integrated approach to the intervention of the potential risk factors for patients with SS. Early diagnosis of SS and initiation of treatment for these patients may prevent the development of RF in patients with pulmonary SS, thereby preventing the need for ICU admission.
